# Axial MRI biomarkers of spinal cord damage to predict future walking and motor function: a retrospective study

**DOI:** 10.1038/s41393-020-00561-w

**Published:** 2020-10-06

**Authors:** Andrew C. Smith, Stephanie R. Albin, Denise R. O’Dell, Jeffrey C. Berliner, David Dungan, Mitch Sevigny, Christina Draganich, James M. Elliott, Kenneth A. Weber II

**Affiliations:** 1 Department of Physical Medicine and Rehabilitation, Physical Therapy Program, University of Colorado School of Medicine, Denver, CO USA; 2 Regis University School of Physical Therapy, Denver, CO USA; 3 Craig Hospital, Englewood, CO USA; 4 Radiology Imaging Associates, Denver, CO USA; 5 Faculty of Medicine and Health, The University of Sydney, Northern Sydney Local Health District, The Kolling Research Institute, St Leonards, NSW Australia; 6 Department of Anesthesiology, Perioperative and Pain Medicine, Stanford University School of Medicine, Palo Alto, CA USA

**Keywords:** Prognostic markers, Prognostic markers

## Abstract

**Study design:**

Retrospective.

**Objectives:**

Primary: to assess if axial damage ratios are predictors of future walking after spinal cord injury (SCI), and if they add any predictive value if initial neurological impairment grades are available. Secondary: to determine if lateral spinal cord regions are predictors of future lower extremity motor scores (LEMS).

**Setting:**

University/hospital.

**Methods:**

Axial T_2_-weighted MRIs were used. Axial damage ratios and non-damaged lateral cord volumes were calculated. Each participant answered at 1 year after SCI, “Are you able to walk for 150 feet? (45.72 meters)” For the secondary aim, right and left LEMS were used.

**Results:**

In total, 145 participants were selected. Individuals that could walk had smaller ratios than those that were unable. Walking and axial damage ratios were negatively correlated. A 0.374 ratio cut-off showed optimal sensitivity/specificity. When initial neurological grades were used, axial damage ratios did not add predictive value. Forty-two participants had LEMS available and were included for the secondary aim. Right cord regions and right LEMS were positively correlated and left regions and left LEMS, but these variables were also correlated with each other.

**Conclusions:**

Axial damage ratios were significant predictors of walking ability 1 year after SCI. However, this measure did not add predictive value over initial neurological grades. Lateral cord regions correlated with same-side LEMS, but the opposite was also found, calling this biomarker’s specificity into question. Axial damage ratios may be useful in predicting walking after SCI if initial neurological grades are unavailable.

**Sponsorship:**

This research was funded by a National Institutes of Health award, National Institute of Child Health and Development—NIH R03HD094577.

## Introduction

Early after a spinal cord injury (SCI), the recovery of walking ability is among the top priorities for individuals with SCI [1]. Clinical prediction rules have been developed to determine who will walk again following SCI [2–4]. An important factor driving these clinical prediction rules is the extent of residual sensorimotor function, which is used to classify patients into American Spinal Injury Association Impairment Scales (AIS) [5]. Unsurprisingly, those with motor incomplete injuries who have extensive voluntary residual motor function in their lower extremities receive a favorable prognosis for walking recovery [2–4].

The clinical prediction rules for predicting walking after SCI have limitations. These tools are not as accurate in predicting walking for those with more severe, but incomplete SCIs (AIS B and C) where prediction would be most clinically useful [6]. Early after SCI, sensorimotor testing for proper AIS classification may not be possible to perform—in instances of sedation, polytrauma, and/or concomitant brain injury—thus, the clinical prediction rules would not be available or applicable. In these instances, magnetic resonance imaging (MRI) biomarkers may be especially useful to aide in the prediction of future walking ability [7, 8].

The above studies measured the length of signal hyperintensity using sagittal T_2_-weighted MRI and related this measure to walking ability at the time of hospital discharge [7, 8]. A number of other studies confirm that hyperintensity length is predictive of future neurological status [7, 9–15]. Yet, there are a lack of studies investigating if SCI imaging characteristics are predictive of long-term walking ability, 1 year or more following injury.

**Fig. 1:**
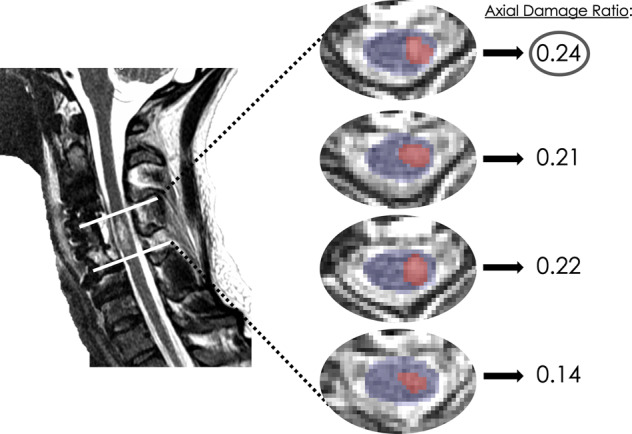
A representative participant’s T_2_-weighted MRI. The left panel shows the mid-sagittal image with the lesion hyperintensity observed. The middle panel shows consecutive axial slices through the damage, with the lesion hyperintensity segmented in red and the surrounding spinal cord segmented in blue. The calculated axial damage ratios are seen in the right panel, with the cranial-most axial slice demonstrating the maximum axial damage ratio used for statistical analysis.

**Fig. 2:**
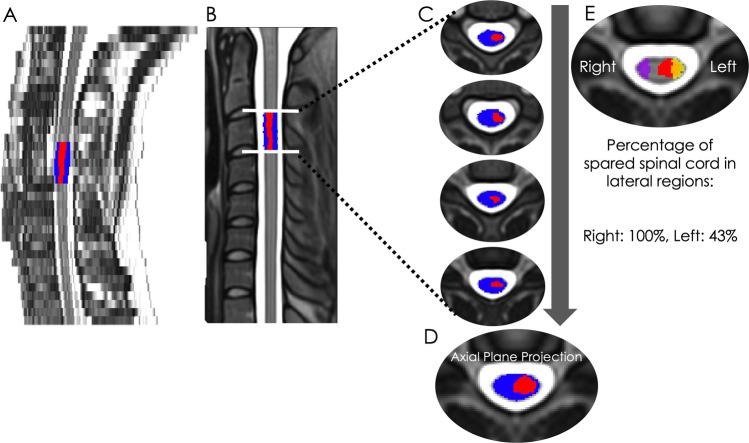
The same representative participant from Fig. 1 is depicted here. The T_2_-weighted image is seen as shown with the spinal cord and lesion masks overlaid (**a**) and then registered to the PAM50 T_2_-weighted template (**b**). To quantify the spatial extent of the spinal cord lesion (**c**), the lesion mask was projected to the axial plane (**d**), and the percentage of spared left or right lateral region volume (non-lesion) within the axial plane was calculated (**e**).

Recently, using axial T_2_-weighted sequences and quantifying the signal hyperintensity after SCI, two novel MRI biomarkers have been reported: (1) axial damage ratio, (2) region-specific damage [16, 17]. The axial damage ratio is determined by finding the axial slice where the area of cord hyperintensity is the largest in relationship to its surrounding cord diameter and calculating the ratio of hyperintensity area divided by cord area (see Fig. 1) [16]. In a cohort of individuals with chronic motor incomplete SCI, the axial damage ratio was significantly related to their performance on the 6-min walk test [16]. For the region-specific damage biomarker, the T_2_-weighted images are registered to a standard spinal cord template image with a corresponding white matter atlas, permitting estimation of damage to specific regions such as the lateral corticospinal tract (see Fig. 2) [17]. In the same cohort as above, damage to the left or right lateral corticospinal tract was significantly related to the ability to generate maximum voluntary torque in the ipsilateral lower extremity [17].

While these results are promising, these two axial MRI biomarker studies used a cross-sectional design involving participants with chronic motor incomplete SCI, 6 years post injury on average. Whether or not the axial MRI biomarkers have potential predictive value for long-term future walking and motor function in a more heterogenous population of persons with SCI is unknown [16, 17]. Additionally, it is unknown whether or not axial MRI biomarkers add predictive value for walking if initial AIS grades are clinically available. Accordingly, the primary aim of this study was to determine if axial damage ratios are significant predictors of walking ability 1 year after SCI, and if they add any predictive value if initial AIS grades are available. The secondary aim of this study was to determine if spared tissue in the lateral cord regions may be significant predictors of future lower extremity motor scores (LEMS).

## Methods

This was a retrospective study involving participants selected from the United States SCI Model Systems database at Craig Hospital. This study was approved by two local Institutional Review Boards. Inclusion criteria were: status post cervical SCI, clinical MRIs available for analyses, and completed enrollment in the Craig Hospital SCI Model Systems between the years of 2010 and 2017 with 1-year outcomes data available. Exclusion criteria were: concurrent traumatic brain injury beyond concussion, and significant preexisting neurological history (i.e., multiple sclerosis, cerebrovascular stroke, etc.). We certify that all applicable institutional and governmental regulations concerning the ethical use of human volunteers were followed during the course of this research.

### Spinal cord magnetic resonance imaging

Routine clinical T_2_-weighted MRIs were used for analysis, acquired postoperatively after SCI (average = 3.7 weeks post SCI). Thirty-two axial images of the cervical spinal cord were acquired using a General Electric 1.5 T Signa Excite MR Scanner equipped with the eight-channel cervical–thoracic–lumbar spine array coil, and a two-dimensional fast relaxation fast spin echo sequence (slice thickness = 3 mm, slice spacing = 4 mm, field-of-view = 200 × 200 mm^2^, matrix size = 224 × 224, in-plane resolution = 0.83 mm^2^, interpolated in-plane resolution = 0.39 × 0.39 mm^2^).

All MRI measurements were completed by an experienced researcher, supervised by a neuroradiologist, who were both blinded to the clinical outcomes of the participants. For the axial damage ratio biomarker, the spinal cord lesion hyperintensity was segmented throughout each axial slice, followed by the surrounding spinal cord, using FSLview (Oxford Center for fMRI of the Brain, University of Oxford, Oxford, UK). The axial damage ratio was calculated for each slice as Area_lesion_/Area_cord_, and the maximum ratio was selected for each participant (Fig. 1). This method was demonstrated to have high inter- and intra-rater reliability [16, 18].

For the region-specific damage biomarker, the open-source Spinal Cord Toolbox (Version 4.0.1) was used to calculate the volume of spared white matter tracts [19]. The toolbox contains a comprehensive set of functions for the processing spinal cord MRI datasets and the PAM50 spinal cord template (resolution = 0.5 × 0.5 × 0.5 mm^3^) with a corresponding probabilistic atlas of 15 pairs of white matter tracts [19–21]. The white matter atlas was registered to the participants’ images using the same segmented masks as the axial damage ratio biomarker. The PAM50 T_2_-weighted template was then registered to the T_2_-weighted image (reference = subject) using the manually drawn spinal cord to mask and the PAM50 template spinal cord mask to perform the registration. Using the segmentation masks prevented the registration from being influenced by the lesion hyperintensity. The vertebral level at the center of the spinal cord lesion was used to localize the registration along the superior–inferior axis. The spinal cord segmentations were then initially aligned using their center of mass, and then a series of non-linear deformations (algorithm = bsplinesyn) were performed to register the template spinal cord mask to the manually draw spinal cord mask. The final registration step included columnwise registration, which allows greater deformation and is recommended for compressed or distorted cords. The corresponding transformation was then used to warp the lesion mask image to the PAM50 template. To quantify the spatial extent of the spinal cord lesion, the lesion mask was projected to the axial plane, and the percentage of spared left or right lateral region volume (non-lesion) within the axial plane was calculated for each region (Fig. 2). The left and right percent spared tissue values were used for statistical analyses. The template registration was visually inspected for quality control.

### Outcome measures: walking ability and lower extremity motor scores

For the primary aim of this study, as part of the United States SCI Model Systems database at Craig Hospital protocol, each participant answered yes/no at 1-year follow up after SCI to the question, “Are you able to walk (with or without mobility aid) for 150 feet (45.72 meters) in your home?” Responses were assigned a binary code of either 1 (yes) or 0 (no).

For the secondary aim of this study, International Standards for the Neurological Classification of Spinal Cord Injury (ISNCSCI) LEMS for the right and left lower extremities were used, collected at the time of discharge from inpatient therapy. The ISNCSCI examinations were completed and agreed upon by pairs of licensed physical and occupational therapists who were extensively trained in ISNCSCI testing, and each report was reviewed and approved by the corresponding team physician. The LEMS clinical testing, assessed using an ordinal scale, has demonstrated high levels of inter-rater reliability [22].

### Statistical analyses

All analyses were performed using SPSS Version 26.0 statistical software (IBM Corporation, Armonk, NY). Descriptive statistics were calculated for demographic, anthropometric, and imaging variables.

### Primary aim

Axial damage ratio descriptive statistics were calculated for all participants, and separately for individuals that were and were not able to walk 150 feet (45.72 meters). Based on 1000 bootstrap samples, 95% confidence intervals were estimated. Statistical comparisons of axial damage ratios were made between the walking and non-walking groups using analysis of covariance with age as a covariate. Findings were considered statistically significant when *p* < 0.05.

Bivariate associations between the self-reported ability to walk and baseline variables were assessed using correlation analyses (point biserial correlations for dichotomous variables and Pearson product moment correlations for continuous variables). Associations were considered statistically significant if *p* < 0.05.

A receiver operator characteristic (ROC) curve was used to analyze the walking outcome. Sensitivity and specificity values were calculated for each level of the predictor variable and plotted on a ROC curve. The point on the curve that was nearest the upper left corner represented the value with the best diagnostic accuracy, and this point was chosen as the cut-off defining the ability to walk. Using the cut-off point generated from the ROC curve, a new dichotomized variable was created and each score was characterized as above or below the cut-off point. Logistic regression was run to assess if the axial damage ratio was a significant predictor of self-reported ability to walk 150 feet (45.72 meters) 1 year after SCI. The following were calculated: sensitivity, specificity, and positive and negative likelihood ratios.

Logistic regression was performed to determine if axial damage ratios added any predictive value for walking versus initial AIS grades alone. In model 1, initial AIS grades were used as a predictor variable and compared to model 2, where both initial AIS grades and axial damage ratios were used as predictor variables.

### Secondary aim

For the investigation of lateral cord region-specific damage, the percent volume of the spared region was used for analysis. Bivariate associations between lateral cord region spared tissue and LEMS were assessed using Spearman correlation analyses. Based on 1000 bootstrap samples, 95% confidence intervals were estimated. Associations were considered statistically significant if *p* < 0.05.

## Results

### Primary aim

One hundred and forty-five participants met the established criteria and their records were used for this study. The baseline demographics are summarized in Table 1. The earliest axial T_2_-weighted imaging used for analyses ranged from 0 to 12 weeks post SCI (average 3.7 ± 2.8 weeks). The self-reported ability to walk at 1 year and the axial damage ratios are reported in Table 2. Individuals that were able to walk 150 feet (45.72 meters) 1 year after SCI had a smaller axial damage ratio than those that were unable to walk (mean difference = 0.124, SED = 0.032, 95% CI: 0.06, 0.188, *p* < 0.001, see Table 2).

**Table 1:** Baseline demographics^a^.

Characteristic	
Age, years	42.1 (17.05)
Sex (male), *n* (%)	121 (83.4)
BMI, kg/m^2^	25.0 (4.9)
Weeks from injury to imaging	3.7 (2.8)
Weeks in inpatient rehabilitation	12.9 (5.6)
Able to walk 150 feet (45.72 meters) (yes), *n* (%)	53 (36.6)

*BMI* body mass index.

^a^Values are mean (SD) unless otherwise indicated.

**Table 2:** Axial damage ratio of all participants and by ability to walk 150 feet (45.72 meters).

	Mean (95% CI)	Standard deviation (95% CI)
All participants (*n* = 145)		
Axial damage ratio	0.47 (0.44, 0.51)	0.19 (0.17, 0.21)
Able to walk (*n* = 58)		
Axial damage ratio	0.37 (0.32, 0.42)	0.18 (0.14, 0.22)
Not able to walk (*n* = 92)		
Axial damage ratio	0.53 (0.49, 0.57)	0.17 (0.15, 0.19)

Self-reported ability to walk was significantly negatively correlated with the axial damage ratio (*R* = −0.404; 95% CI: −0.537, −0.258, *p* < 0.001). Age and the ability to walk at 1 year were significantly positively correlated (*R* = 0.397; 95% CI: 0.251, 0.539, *p* < 0.001) Age at the time of injury and the axial damage ratio were significantly negatively correlated (*R* = −0.372; 95% CI: −0.507, −0.230, *p* < 0.001).

For the ROC curve analysis, with the state variable being the ability to walk 150 feet (45.72 meters) at 1 year after SCI and the test variable the axial damage ratio, it was found that the area under the curve (AUC) was 0.751 (95% CI: 0.667, 0.834, *p* < 0.001). An axial damage ratio cut-off value of 0.374 was chosen based on the position of the upper left corner of the curve with consideration of optimizing of specificity. Another variable was created dichotomizing individuals above and below this cut-off point. The logistic regression model for the axial damage ratio below 0.374 was a significant predictor of the ability to walk 1 year after injury (*Χ*^2^ = 20.62, *p* < 0.001, Nagelkerke *R*^2^ = 0.181, Exp(*B*) = 5.36; 95% CI: 2.54, 11.34). The sensitivity of using the axial damage ratio cut-off of 0.374 was 0.57 and the specificity was 0.80. The positive likelihood ratio was 2.89 (95% CI: 1.80, 4.66) and the negative likelihood ratio was 0.54 (95% CI: 0.39, 0.75).

The logistic regression model for initial AIS grades showed this variable to be a significant predictor of the ability to walk 1 year after injury (*Χ*^2^ = 111.91, *p* < 0.001, *R*^2^ = 0.743). When axial damage ratios were added as an additional predictor, this variable was non-significant (*p* = 0.783).

### Secondary aim

As a subset of the participants included in the primary aim, 42 participants had right and left LEMS available and were included for the secondary aim analysis. Average length of inpatient stay was 13.2 ± 6.5 weeks. The lateral cord region percent spared tissue volumes and the LEMS are reported in Table 3. There was a significant positive correlation with right lateral cord region percent spared tissue and right LEMS (*R*s = 0.654, 95% CI: 0.440, 0.813, *p* < 0.0001) and with left lateral cord region spared tissue and left LEMS (*R*s = 0.558, 95% CI: 0.323, 0.741, *p* < 0.0001).

**Table 3:** LEMS and lateral cord percent spared tissue (subset of *N* = 42 participants).

Right LEMS	Left LEMS	Right lateral cord % spared	Left lateral cord % spared
4.62 (6.61)	5.98 (8.34)	36.42 (32.73)	36.17 (31.34)

*LEMS* lower extremity motor scores.

## Discussion

We found that the axial damage ratio taken from MRIs early after injury was a significant predictor of walking ability 1-year post SCI, meaning the smaller the damage ratio, the more likely the individual reported “yes” for ability to walk 150 feet (45.72 meters) at 1 year. This finding is in accordance with a previous incomplete SCI cohort study, where axial damage ratios correlated with performance on the 6-min walk test [16]. Interestingly, we found a significant positive relationship between age and walking ability, meaning the older the individual was at the time of injury, the more likely the patient was able to walk 1-year post injury. When we explored this further, we found a significant negative correlation between age at the time of injury and the axial damage ratio, which suggests that, in this study’s cohort, younger individuals had greater axial damage ratios. We believe this is due to the nature of Craig Hospital, where relatively younger patients admitted to inpatient rehabilitation tend to have more severe SCIs compared to the older patients. Per previous literature, older age has been shown to be a negative predictor of future walking ability [2, 3].

Our axial damage ratio ROC curve had an AUC of 0.75, indicating fair discriminative ability for determining walking 1 year after SCI [23]. A previous study using a lesion length of 36 mm found a similar AUC value (0.776) for discriminating neurological outcome [15]. The axial damage ratio cut-off score for our cohort was 0.374, with ratios below this significantly associated with a “yes” response to walking. Previous literature examined the associations between future function after SCI and the extent of T_2_-weighted MRI-observed lesion hyperintensity in the sagittal plane [7, 13]. One study found that participants with incomplete SCIs had lesion length of ~20 mm while those with complete SCIs had up to 40 mm [13]. Another study reported that individuals with AIS D injuries tended to have a lesion length of less than 45 mm, and these patients were able to walk with or without a cane at discharge [7]. A third study that included 86 patients with incomplete SCI found that a lesion length of greater than 36 mm was independently associated with a more severe AIS grade 1 year later [15]. The sensitivity of our axial damage ratio cut-off score was relatively lower (0.57) compared with the specificity (0.80). This suggests that this biomarker, early after SCI, might serve best for ruling in those who have good potential for future walking, rather than ruling out.

For our region-specific damage biomarker, the percentage of spared tissue for the right lateral cord was correlated with right LEMS, and the same was true for the left side. This result is in alignment with our previous cross-sectional study, using a higher resolution outcome measure of isometric maximum voluntary torque production of the knee extensors and ankle plantarflexors [17].

### Limitations

For the purposes of this study, we deliberately wanted to focus on the prognostic value of the axial MRI biomarkers, as it is more clinically obvious that following SCI, existing lower extremity strength will likely lead to some ability to ambulate in the future [24]. Previous research also cites this issue when focusing on imaging biomarkers for prognosis in SCI [8]. Nevertheless, when we included initial AIS grades as a predictor, the inclusion of the MRI measure did not add significant predictive value. This suggests that, similar to a recent previous study, initial AIS grades remain the most optimal predictor, if clinically available [25]. Another limitation is that, due to the nature of our experimental design, our axial damage ratio cut-off score of 0.374 is specific to our cohort, and these results need to be validated in additional independent cohorts before an axial damage ratio cut-off score can be generalizable to the population with SCI as a whole. An inherent limitation of retrospective studies is that researchers are limited to data available for analyses, and for this study, the earliest imaging available averaged 3.7 ± 2.8 weeks. This is a possible confounder, as SCI imaging characteristics are dynamic, especially within the first 3 weeks of injury [26]. Although only one blinded researcher performed the measures in this study (which is a limitation), we are confident in the results as evidenced by reported high levels of inter-rater reliability with the same measures [11]. Lastly, only a subset of our cohort (42 out of 145) had discharge LEMS scores to use for analysis in our region-specific damage secondary aim, and only ten of those participants showed clinically meaningful asymmetries in their residual motor function. There is also a question of the specificity of this biomarker given that the left and right lateral cord spared tissue were significantly correlated with each other in the current study (*R* = 0.829, *p* < 0.05). We also found that right lateral cord percent spared tissue was significantly correlated with left LEMS, and vice versa (left cord versus right LEMS *R* = 0.569, and right cord versus left LEMS *R* = 0.620, *p* < 0.05). Future prospective research should focus on a larger sample with these data available, and should examine this issue of specificity of the region-specific damage biomarker.

## Conclusion

In this retrospective study involving 145 participants with SCI, T_2_ MRI axial damage ratios early after injury demonstrated significant predictive value for self-reported ability to walk 150 feet (45.72 meters) 1-year after SCI, and the ROC curve had an AUC value of 0.75, indicating fair discriminative ability. However, when including initial AIS grades as a predictor, axial damage ratios did not add significant additional predictive value. In a subset of 42 participants, lateral cord region spared tissue was correlated with the same-side LEMS, but the opposite was also found, calling the specificity of the biomarker into question. The axial damage ratio biomarker may be a useful tool in predicting future walking ability after SCI, but initial AIS grades remain the optimal predictor if clinically available. Future research is warranted to prospectively validate these findings.

## Data Availability

The dataset analyzed during the current study are available from the corresponding author on reasonable request.
